# Spatio-temporal evolution of the COVID-19 across African countries

**DOI:** 10.3389/fpubh.2022.1039925

**Published:** 2022-11-28

**Authors:** Bechir Naffeti, Sebastien Bourdin, Walid Ben Aribi, Amira Kebir, Slimane Ben Miled

**Affiliations:** ^1^Laboratory of BioInformatics, bioMathematics and bioStatistics, Institute Pasteur of Tunis, Tunis, Tunisia; ^2^Métis Lab, EM Normandie Business School, Le Havre, France; ^3^Preparatory Institute for Engineering Studies of Tunis, University of Tunis, Tunis, Tunisia

**Keywords:** reproduction number *R*_0_, epidemiology, Africa, regional analysis, COVID-19, SIR model, SARS-CoV-2

## Abstract

The aim of this study is to make a comparative study on the reproduction number *R*_0_ computed at the beginning of each wave for African countries and to understand the reasons for the disparities between them. The study covers the two first years of the COVID-19 pandemic and for 30 African countries. It links pandemic variables, reproduction number *R*_0_, demographic variable, median age of the population, economic variables, *GDP* and *CHE* per capita, and climatic variables, mean temperature at the beginning of each waves. The results show that the diffusion of COVID-19 in Africa was heterogeneous even between geographical proximal countries. The difference of the basic reproduction number *R*_0_ values is very large between countries and is significantly correlated with economic and climatic variables *GDP* and temperature and to a less extent with the mean age of the population.

## 1. Introduction

On January 30, 2020, the World Health Organization (WHO) declared COVID-19 as a Public Health Emergency of International Concern^1^ and by March 11, 2020, declared the first pandemic caused by the coronavirus. Up to July 2021, COVID-19 has affected over 187 million people with more than 4 million associated deaths and in addition, has induced catastrophic public health and socio-economic affliction globally (1).

**Figure 1:**
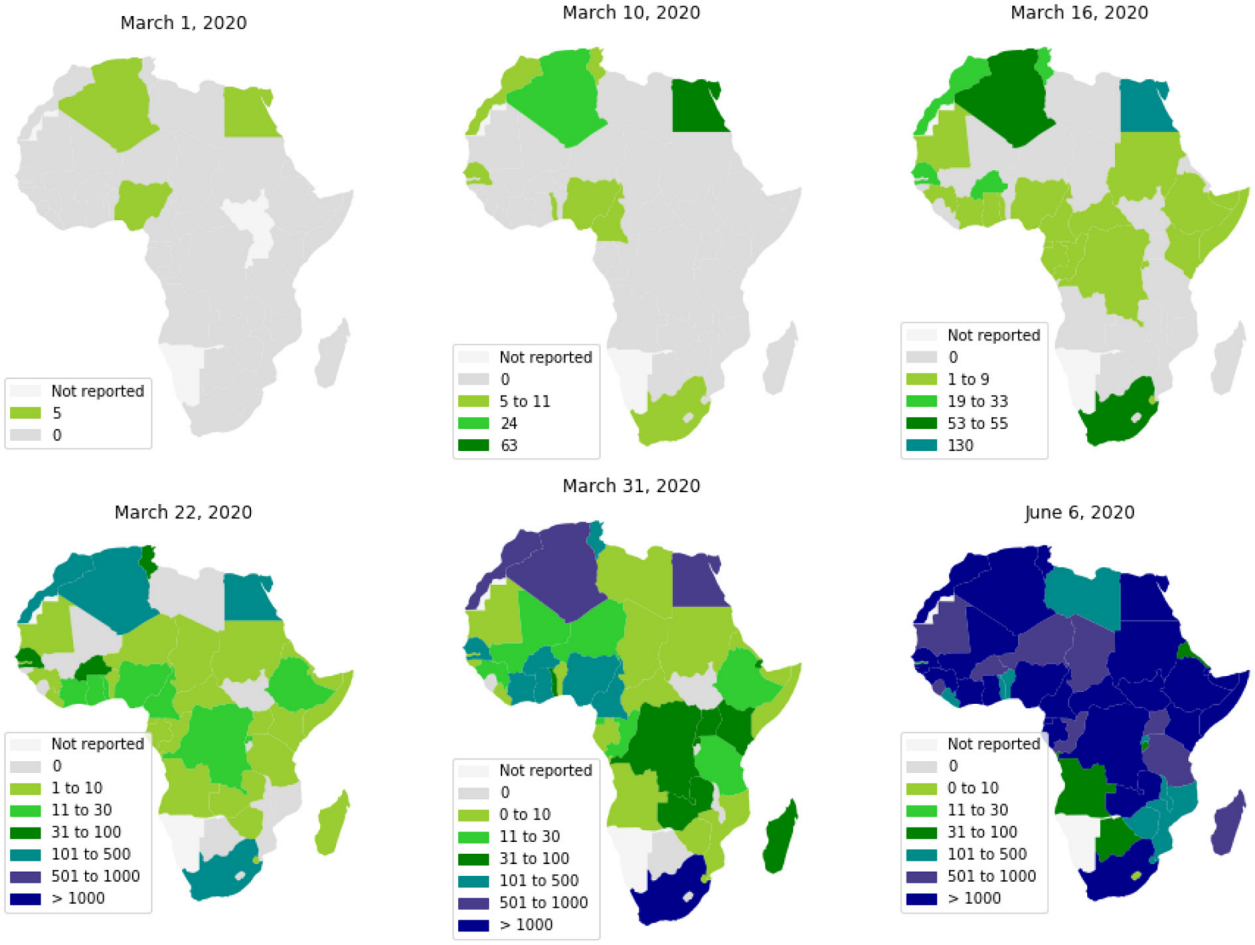
Spread of COVID-19 in Africa.

The first cases in Africa to be reported by WHO were respectively, on February 14, 18, and 25, 2020, in Egypt, Algeria, and Nigeria. These first cases have nearly coincided with those in Europe, which is likely the original source of pathogen introduction in Africa.^2^ Since then, the virus has spread quite quickly (see Figure 1) (2). Up to June 6, 2020, most African countries have crossed the threshold of 1, 000 cases and the whole continent had 175, 423 cumulative cases and 4, 862 reported deaths. The WHO had predicted that 29 to 44 million Africans would be infected with COVID-19 during the first year of the pandemic, and 83 to 190 thousand Africans would had die if they don't uphold containment measures.^3^

The high levels of poverty, weak health systems, and a large number of crowded urban areas, make the virus particularly devastating in African countries.^4^ However, the warmer climate, the population youth, and the boosted immunity by long exposure to previous endemic pathogens, would allow the continent to mitigate the risk of the pandemic (3). In this context, the diversity of COVID-19's dynamics throughout Africa and its relationship to socioeconomic and environmental factors can help us better understand the epidemic's determinism.

Like European countries (4), at the beginning of the COVID-19 epidemic, most African countries implemented strict Non-Pharmaceutical intervention (NPI) to limit the spread of this pandemic (5–7). This has included: the obligation to mask wearing and social distancing measures at the individual level, frontier closure, the closure of schools, universities, and public places, the closure of mosques and churches, and the prohibition of movement between cities and provinces. These measures have contributed in reducing the spread of the pandemic (8, 9). However, considering the socio-economical heterogeneity of the African countries (10), the response to these measures differed from one country to another as evidenced by the disparities between regions in infected cases and wave numbers (11).

To date, few studies have analyzed how the pandemic spread in Africa and how its intensity varied over time (12–16). Moreover, to our knowledge, no study has been conducted to analyze what are the determinants that could explain the geography of the pandemic.

This study aims at analyzing the Spatio-temporal evolution of the COVID-19 infection across 30 African countries and for each wave until March, 2022. And to provide demo-economical and environmental factors that can better explain the regional heterogeneity of the basic reproduction rate, *R*_0_. To this end, we calculate *R*_0_ at the early beginning of each wave, in order to avoid taking into account the NPI measure. We then make a correlation analysis between *R*_0_ and collected demographic, economic, and climatic data so as to assess how these factors may account for the regional variations of the pandemic.

The document is organized as follows: In Section 2, the material and method are presented. In Section 3, results and discussion are given. Finally, the conclusion is given in Section 4.

## 2. Materials and methods

In order to comprehend the differences between African countries, we collected epidemiological data from 45 African countries. Due to the quality of the data, this list was reduced to 30 countries distributed between North, South, East and West Africa. These countries are: Algeria, Angola, Burkina Faso, Cameroon, Chad, Ivory Coast, Egypt, Ethiopia, Guinea, Guinea-Bissau, Kenya, Libya, Madagascar, Mali, Mauritania, Morocco, Mozambique, Namibia, Niger, Nigeria, RDC, Rwanda, Senegal, Somalia, South Africa, Sudan, Tanzania, Tunisia, Zambia, and Zimbabwe.

Up to March, 2022, With the exception of Tanzania, Madagascar, Chad, and Burkina Faso, which had three waves, and Kenya, Algeria, Tunisia, and Zambia, which had five waves, nearly all of the thirty African countries analyzed had four waves. For all countries, the Omicron variant generated the most recent wave.

We took into account six epidemiological, demo-economical and climate factors for each country:

- Epidemiological variables are: The basic reproduction numbers, *R*_0_, of each wave, used to analyze the temporal evolution of the COVID-19 wave by wave at each country. The second one is the mean value of *R*_0_ over waves, denoted by *MeanR* where $MeanR=\frac{1}{n}\overset{n}{\underset{i=1}{\sum }}R_{0}^{i}$, $R_{0}^{i}$ is the *R*_0_ of the wave *i* and *n* is the number of waves. the *MeanR* is used to for an inter-countries comparison.
- Economic variables: The current health expenditure (CHE), and the gross domestic product (GDP) were collected from World Bank data.^5^ It has been shown that these variables have an impact on the propagation of the pandemic in several countries (17–19).
- Climate variable: Mean of the country's temperature at the periods of the beginning waves.^6^,^7^
- Demographic variable: The median of ages of the population population (see text footnote 5) as older patients are at higher risk of developing severity (20).

For each wave and each African country, basic reproduction rate, *R*_0_, was computed using the method developed in (21). This method is based on a SIR model, which is an Ordinary Differential Equations (ODE) that describes a structured population through three classes: S (susceptible), I (infected: reported and unreported), and R (removed: recovered or die). For more detail about the system of ordinary differential equations, parameters identification and how to determine the *R*_0_ values see Appendix. As it is difficult to estimate the impact of control policies in the calculation of *R*_0_, we chose to calculate *R*_0_ with data from the first days of each wave. Indeed, we assume that at the beginning of each wave the control policies are very little applied or non-existent, so the growth of the pandemic is exponential.

To measure the degree of the relationship between variables, we use the Pearson correlation defined by (22).

For the clustering countries with similar data variables, we use an “unsupervised learning” method, the hierarchical clustering (23).

In this method, it is not necessary to specify an initial number of clusters to run the algorithm. Dendrogram was used to visualize the partitioning of the data.

Impact data variables were summarized and visualized using Principal Component Analysis (PCA) (24).

**Figure 2:**
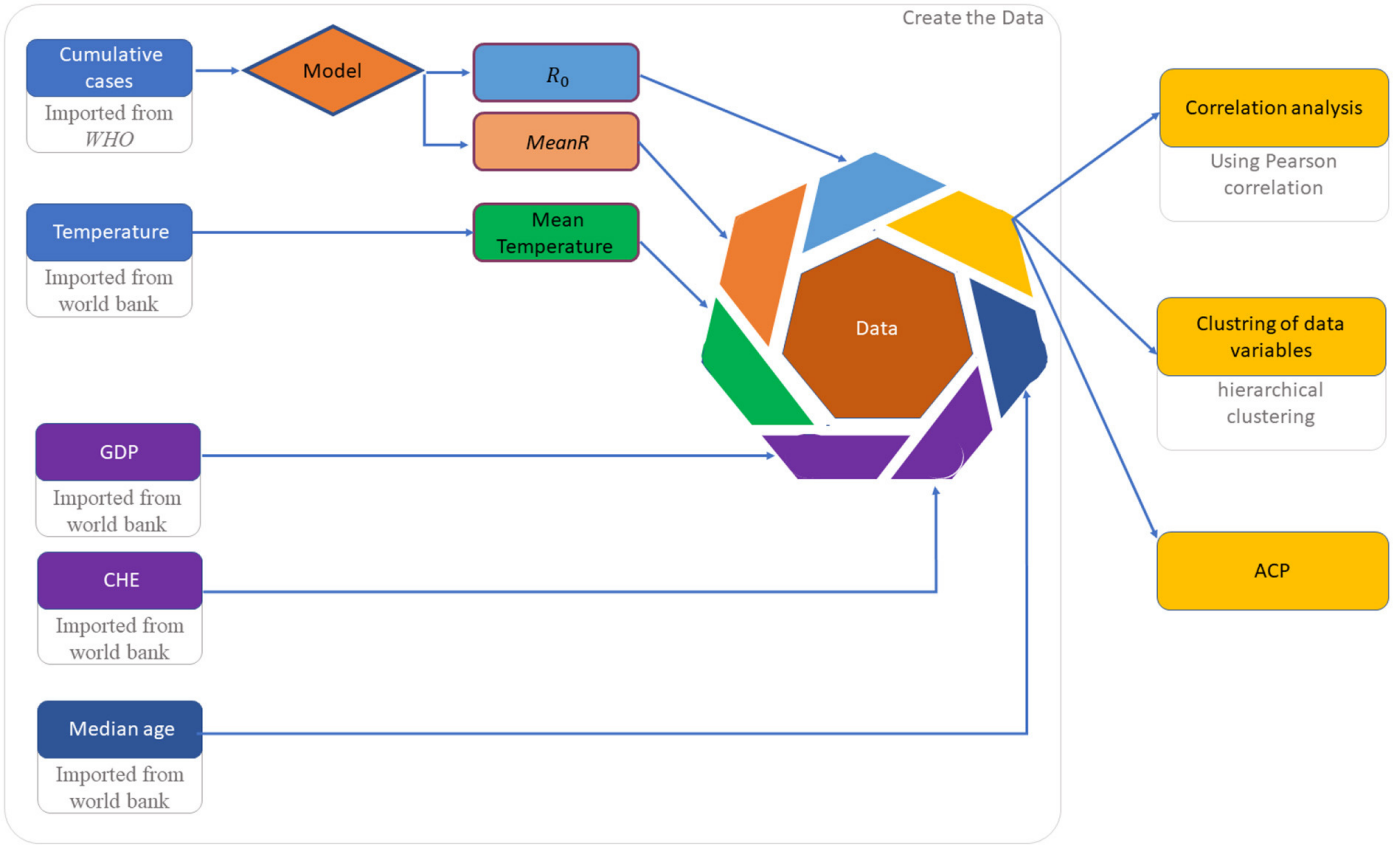
Flowchart diagram to explain the different steps of the methodology.

Data set implementation and analysis is described in Figure 2.

## 3. Results and discussion

In this section, we perform inter-country and intra-country analyses integrating economic, climatic, and demographic factors.

### 3.1. Inter-country analysis

**Figure 3:**
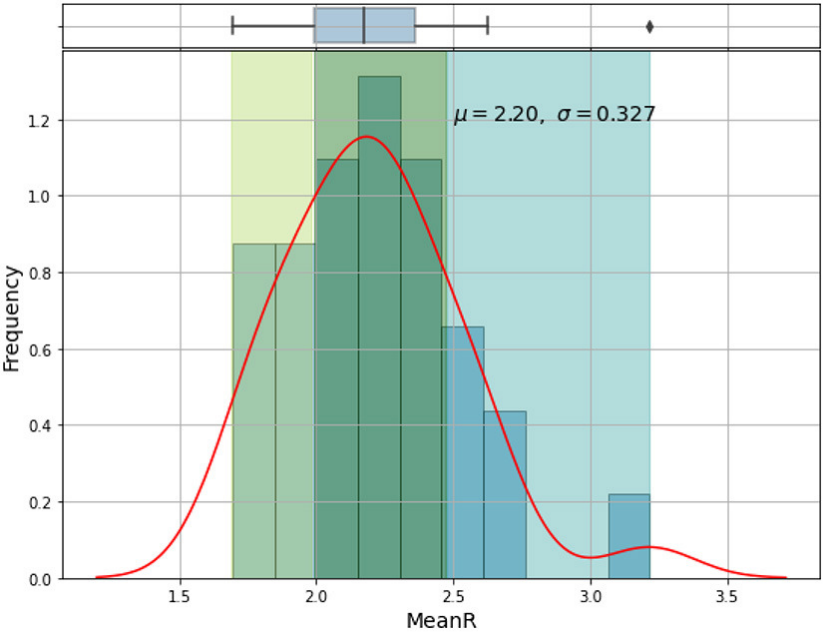
Frequency distribution and histogram of *MeanR* of all waves.

**Figure 4:**
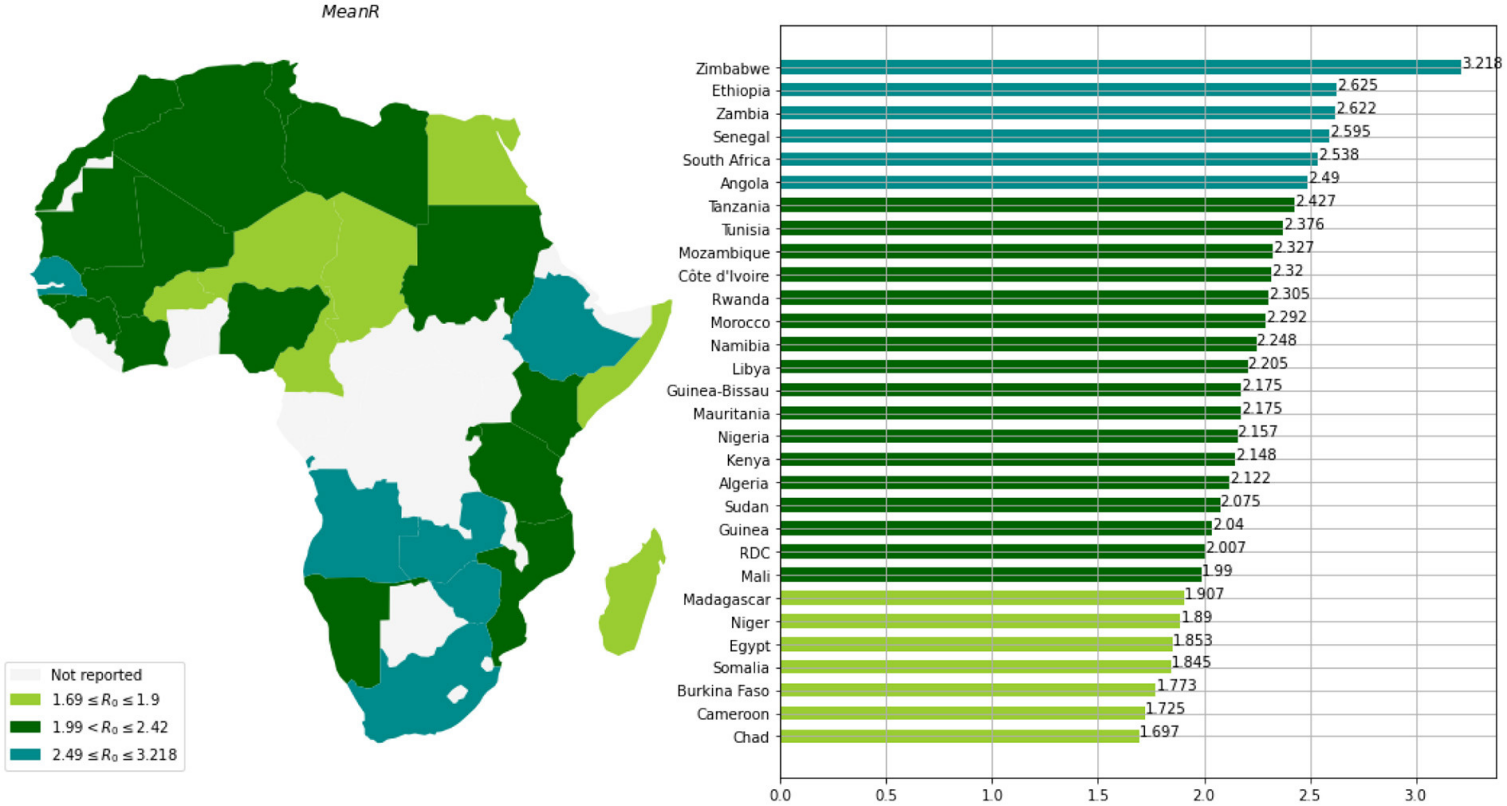
Distribution of *MeanR* among 30 countries in Africa.

Based on the mean of *R*_0_, *MeanR*, distribution across waves (see Figure 3), we divided countries into three groups (see Table 1 and Figure 4).

**Table 1:** Distribution of *MeanR* by Country.

	***MeanR***	**Country**
Group 1	[2.49, 3.22]	Senegal, Zimbabwe, South Africa,
		Angola, Zambia, Ethiopia
Group 2	[1.99, 2.43]	Mali, RDC, Guinea, Sudan, Algeria
		Kenya, Nigeria, Mauritania, Libya
		Guinea-Bissau, Namibia, Morocco
		Rwanda, Côte d'Ivoire, Tunisia
		Mozambique, Tanzania
Group 3	[1.69, 1.907]	Madagascar, Niger, Egypt, Somalia,
		Chad, Burkina-Faso, and Cameroon

We observe that more than 50% of the countries have *MeanR* values in [1.99, 2.37], mainly located in north Africa. Moreover, 25% of the other countries show higher values of *MeanR* and are located especially in South Africa.

### 3.2. Intra-country distribution

When comparing the first three waves, we can see that the *R*_0_ values for waves 4 and 5 have significantly grown (see Table 2). This makes sense given how quickly the Delta and Omicron variants have spread.

**Table 2:** Mean *R*_0_ over countries by waves.

	**First wave**	**Second wave**	**Third wave**	**Fourth wave**	**Fifth wave**
*R*_0_	1.87	2.04	1.91	2.95	3.53

**Figure 5:**
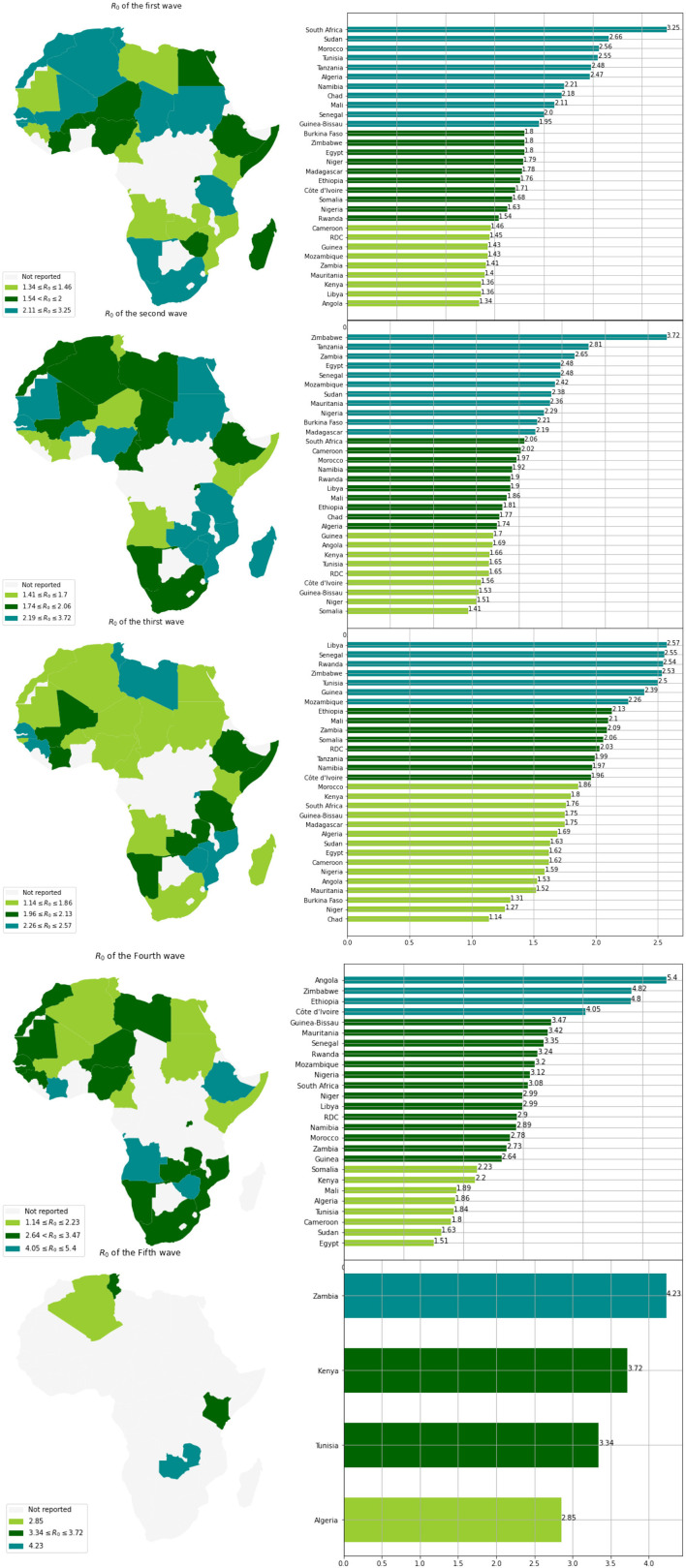
Distribution of the *R*_0_ of 30 countries in Africa for each wave.

Based on the values of *R*_0_ and for each wave, we clustered the countries into three groups (see Table 3 and Figure 5). We observe that countries with the highest *MeanR*, corresponding to Group 1, had experienced a strong first or second wave (countries in Group 1 for the first or second wave).

**Table 3:** Distribution of the country between a Group of *R*_0_ for each wave.

	**First wave**		**Second wave**		**Third wave**		**Fourth wave**		**Fifth wave**	
	**[*R*_0_]**	**Country**	**[*R*_0_]**	**Country**	**[*R*_0_]**	**Country**	**[*R*_0_]**	**Country**	**[*R*_0_]**	**Country**
Group 1	[1.95, 3.25]	Guinea-Bissau	[2.19, 3.72]	Madagascar	[2.26, 2.57]	Mozambique	[4.05, 5.4]	Angola, Zimbabwe	[4.23]	Zambia
		Mali		Burkina Faso		Guinea		Ethiopia, Côte d'Ivoire		
		Senegal		Nigeria		Tunisia
		Chad		Mauritania		Zimbabwe
		Sudan		Sudan		Rwanda
		Namibia		Mozambique		Senegal
		Algeria		Senegal		Libya
		Tanzania		Egypt		
		Tunisia		Zambia		
		Morocco		Tanzania		
		South Africa		Zimbabwe		
Group 2	[1.54, 1.8]	Rwanda	[1.74, 2.06]	Algeria	[1.96, 2.13]	Côte d'Ivoire	[2.64, 3.47]	Guinea-Bissau, Mauritania	[3.34, 3.72]	Tunisia
		Nigeria		Chad		Namibia		Senegal, Rwanda		Kenya
		Somalia		Ethiopia		RDC		Mozambique, Nigeria		
		Côte d'Ivoire		Mali		Somalia		South Africa, Niger		
		Ethiopia		Rwanda		Zambia		Libya, RDC, Namibia		
		Madagascar		Libya		Mali		Morocco, Zambia, Guinea		
		Niger		Namibia		Ethiopia
		Zimbabwe		Morocco		Tanzania
		Burkina Faso		Cameroon		
				South Africa		
Group 3	[1.34, 1.46]	Angola	[1.41, 1.7]	Somalia	[1.14, 1.86]	Mauritania	[1.51, 1.46]	Somalia, Kenya, Mali	[2.85]	Algeria
		Kenya		Niger		Nigeria		Algeria, Tunisia		
		Libya		Guinea-Bissau		Egypt		Cameroon, Sudan, Egypt		
		Mauritania		Côte d'Ivoire		Sudan
		Zambia		Tunisia		Algeria
		Mozambique		RDC		Guinea-Bissau
		Guinea		Kenya		South Africa
		RDC		Angola		Kenya
		Cameroon		Guinea		Morocco
						Madagascar
						Cameroon
						Angola
						Burkina-Faso
						Niger
						Chad

**Groups 1, 2, and 3:** Are the respectively the classes of the highest, the average and the lowest values. **[*R*_0_]:** Is the interval of the *R*_0_ values at each class.

Low *R*_0_ waves were experienced by the countries with the lowest *MeanR* Group (Group 3 for the *MeanR*). Indeed, some of the least affected countries (belonging to Group 2 or 3), have experienced three weak waves, such as Kenya and Guinea (Group 3, for the three waves) or a medium wave as Libya (Group 3 for waves 1 and group 2 for wave 2) RDC (Group 3 for waves 1 and 2 and Group 2 for wave 3) and Mauritania and Cameroon (Group 3 for waves 1 and 3).

We note that, in general, countries in the first Group for the first wave (except for Senegal, Tanzania, and Sudan) experienced a weaker second and third wave. Conversely, countries that experienced a weaker first wave (Groups 2 and 3), experienced a stronger second or third wave (Group 1). Indeed, in Tunisia, the first and third waves (belonging to Group 1), were significant, but the second wave was less so (belonging to Group 3). Senegal experienced three major waves (belonging to Group 1). Finally, South Africa, Chad, Morocco, and Algeria had a powerful first wave (belonging to Group 1), a moderate second wave (belonging to Group 2), and a weak third wave (belonging to Group 3).

### 3.3. Impact of economic factor

Next, we looked at the relationship between the mean *R*_0_ values, *MeanR*, and the Gross Domestic Product (*GDP*), and the Current Health Expenditure (*CHE*) (see Table 4 and data in the Appendix).

**Table 4:** Results of correlation analysis.

**Country**	**Correlation coefficients**
***MeanR*** **vs. *GDP***	
Mozambique, Mauritania, Nigeria Libya, Madagascar, Kenya, South Africa,	0.722
RDC, Chad, Côte d'Ivoire, Sudan, Mali, Niger, Guinea-Bissau, Morocco,	
Tunisia, Somalia, Namibia, Burkina Faso, Guinea, South Africa, Algeria	
***MeanR*** **vs. *CHE***	
Mozambique, Mauritania, Nigeria, Libya, Cameroon, Rwanda, Madagascar,	0.563
South Africa, Guinea, RDC, Chad, Côte d'Ivoire, Sudan, Mali, Morocco,	
Tunisia, Zimbabwe,Kenya, Angola,	
***MeanR*** **vs. Median age**	
Mozambique, Mauritania, Nigeria, Libya, Cameroon, Rwanda, Madagascar,	0.626
South Africa, Guinea, RDC, Chad, Côte d'Ivoire, Sudan, Mali, Niger,	
Morocco, Tunisia, Somalia, Namibia, Burkina Faso,Kenya, Guinea-Bissau,	
***MeanR*** **vs. Mean Temperature**	
Mozambique, Mauritania, Nigeria, Libya, Rwanda, Madagascar, Kenya,	−0.729
Guinea, RDC, Chad, Côte d'Ivoire, Sudan, Mali, Niger, Guinea-Bissau,	
Algeria, Tunisia, Angola, Somalia, Tanzania, Zambia, Namibia,	
Burkina Faso, Morocco, South Africa	

**Country:** The country used for the correlation analysis. **Correlation coefficient:** Is the value of the Pearson coefficient. **MeanR** **vs. Y:** The analysis of the correlation between the variable *MeanR* and *Y*. where *Y*∈{*GDP, CHE, Median*_*age*_, *MeanTemperature*}. We Show that if economic and the age are increasing, the *MeanR* increasing while it is decreasing if the temperature increasing.

**Figure 6:**
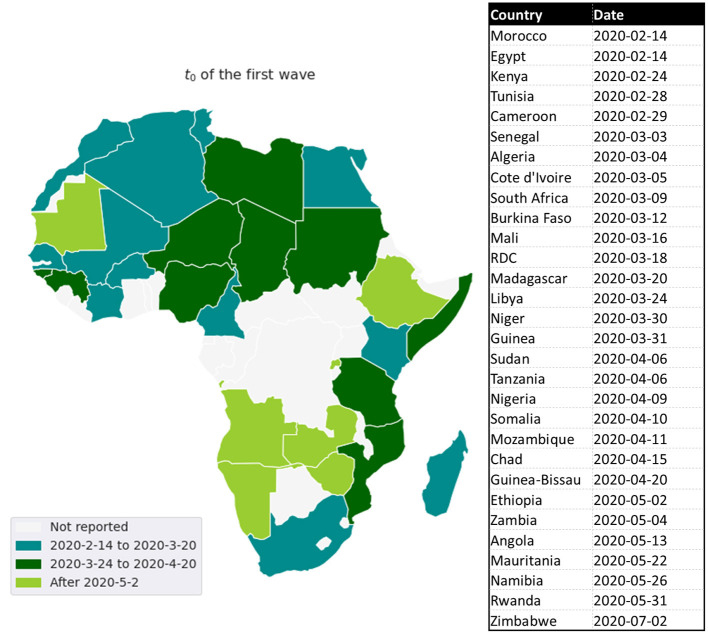
Distribution of the *t*_0_ for the first wave.

It is revealed that the *MeanR* is highly positively correlated to *GDP* and is moderately positively correlated to *CHE*. Indeed, countries with the highest *GDP*s in Africa (*GDP*s above US$3000 per capita), especially South Africa and some North African countries like Tunisia, Morocco, and Algeria, experienced a significant first wave (see Table 5). These countries were the first to be impacted by the epidemic because of their degree of development, which makes them more accessible to international trade (see Figure 6).

**Table 5:** Distribution of the countries between classes waves of *R*_0_ and GDP (in *US$*).

	***GDP*** **>3000**	**1000 < *GDP* ≤ 3000**	***GDP*** **≤ 1000**
**First wave**			
*C*_1_(1)	Morocco, South Africa,	Tanzania,	Sudan, Chad,
	Tunisia, Namibia,	Senegal	Guinea-Bissau
	Algeria,		Mali,
*C*_2_(1)	Egypt,	Zimbabwe,	Somalia, Madagascar,
		Côte d'Ivoire	Rwanda, Burkina-Faso,
			Ethiopia, Niger,
*C*_3_(1)	Libya	Guinea, Cameroon	Mozambique, RDC,
		Angola, Kenya,	Zambia
		Mauritania,	
**Second wave**			
*C*_1_(2)	Egypt	Tanzania, Senegal,	Sudan, Madagascar,
		Zimbabwe, Mauritania	Mozambique, Zambia
			Burkina-Faso,
*C*_2_(2)	Morocco, South Africa,	Cameroon	Chad, Mali,
	Algeria, Namibia,		Rwanda, Ethiopia
	Libya		
*C*_3_(2)	Tunisia	Guinea, Angola,	Guinea-Bissau, Somalia,
		Côte d'Ivoire, Kenya,	Niger, RDC
**Third wave**			
*C*_1_(3)	Libya, Tunisia	Zimbabwe, Guinea	Mozambique, Rwanda,
			Ethiopia
*C*_2_(3)	Namibia	Tanzania, Senegal,	Zambia, Mali,
		Côte d'Ivoire	RDC, Somalia,
*C*_3_(3)	Egypt, Morocco,	Mauritania, Cameroon,	Sudan, Madagascar,
	Algeria, South Africa,	Angola, Kenya	Guinea-Bissau, Niger,
			Burkina-Faso, Chad
**Fourth wave**			
*C*_1_(4)		Zimbabwe, Côte d'Ivoire,	Ethiopia, Guinea-Bissau
		Angola,	
*C*_2_(4)	Libya, Namibia,	Guinea, Senegal	Mozambique, Rwanda,
	South Africa, Morocco,	Mauritania	RDC, Niger
			Zambia,
*C*_3_(4)	Tunisia, Egypt,	Cameroon, Kenya	Mali, Somalia, Sudan
	Algeria		
**Fifth wave**			
*C*_1_(5)			Zambia
*C*_2_(5)	Tunisia	Kenya	
*C*_3_(5)	Algeria		
**MeanR**			
*C*_1_	South Africa	Zimbabwe, Senegal, Angola	Ethiopia, Zambia
*C*_2_	Morocco, Algeria, Tunisia,	Tanzania, Guinea, Kenya,	Mozambique, Sudan,
	Libya	Mauritania, Nigeria,	Guinea-Bissau, Rwanda,
		Côte d'Ivoire	Mali,RDC,
*C*_3_	Egypt	Cameroon	Somalia, Madagascar, Niger,
			Chad, Burkina-Faso

$C_{i}^{\left(j\right)}$**:** is the group *i, i* = 1, 2, 3 at wave *j, j* = 1, 2, 3, 4, 5. We show that countries with the highest GDPs in Africa (GDPs above US3000*$* per capita), especially South Africa and some North African countries like Tunisia, Morocco, and Algeria are experienced a significant first wave.

The lower relationship between *R*_0_ and *CHE* may be explained by two facts: Firstly, we measured *R*_0_ at the start of the wave when public health interventions were either not yet in place or were poorly in place. Secondly, the *CHE* plays a role in terms of preparedness and impact to improve the public health policy between waves in terms of screening capacity. A country with a high *CHE* has the material, human and technological resources to perform the volume of diagnostic tests and thus has the capacity to rapidly identify confirmed cases. This implies a strong dependence between the number of tests and *CHE* (17). It was noted that countries with low health system investment, *CHE*, often have a low testing capacity which makes it difficult to assess the true extent of COVID-19. For example, as of mid-April 2020, the Democratic Republic of the Congo was only performing about 200 tests per day (25), Senegal about 300 tests per day, and Ethiopia about 400 tests per day while the number of tests was 3493 in South Africa.^8^ For these countries, the question of the quality of the data and the reality of the virus circulation arises.

### 3.4. Impact of demographic factors

According to Table 4, there is a correlation between the demographic factors, i.e., the median age and *MeanR*. We observe (see Figure 5), that most countries with a median age under 18 have a *MeanR* less than 2, including Niger, Mali, Chad, Somalia, and Burkina Faso. While South Africa and other countries with a median age greater than 27 have a *MeanR* greater than 2.

This result may be explained by the fact that older people are over-represented in the COVID-19 data since they are more likely to be tested and have more serious infections (26). In contrast, younger people tend to be in better health than older ones, making them more immune to infection. This has been observed in the influenza pandemic in Africa where children and adolescents had a negligible epidemiological impact (27).

### 3.5. Impact of climatic factors

The annual temperature and *MeanR* are negatively highly correlated, as seen in Table 4 and Figure 5. Indeed, we observe that almost all of the countries with a lower value of *meanR* have a dry climate and a high annual temperature (annual temperature greater than 27°C), in contrast to the northern countries, which have a lower annual temperature (annual temperature less than 23°C; see Figure 4).

Note that, negative correlation had already been observed in China (28), in several Latin American countries (29, 30), in the U.S.A. (31) and in Japan (32). From a biological point of view, low humidity dries out the nasal mucosa and impairs the stability of the aerosol droplets and therefore virus particles (33). Hence, the virus replication is limited by temperatures (20 and 30°C) (34).

### 3.6. Clustering of African country from epidemic, economic, demographic, and climatic variables

**Figure 7:**
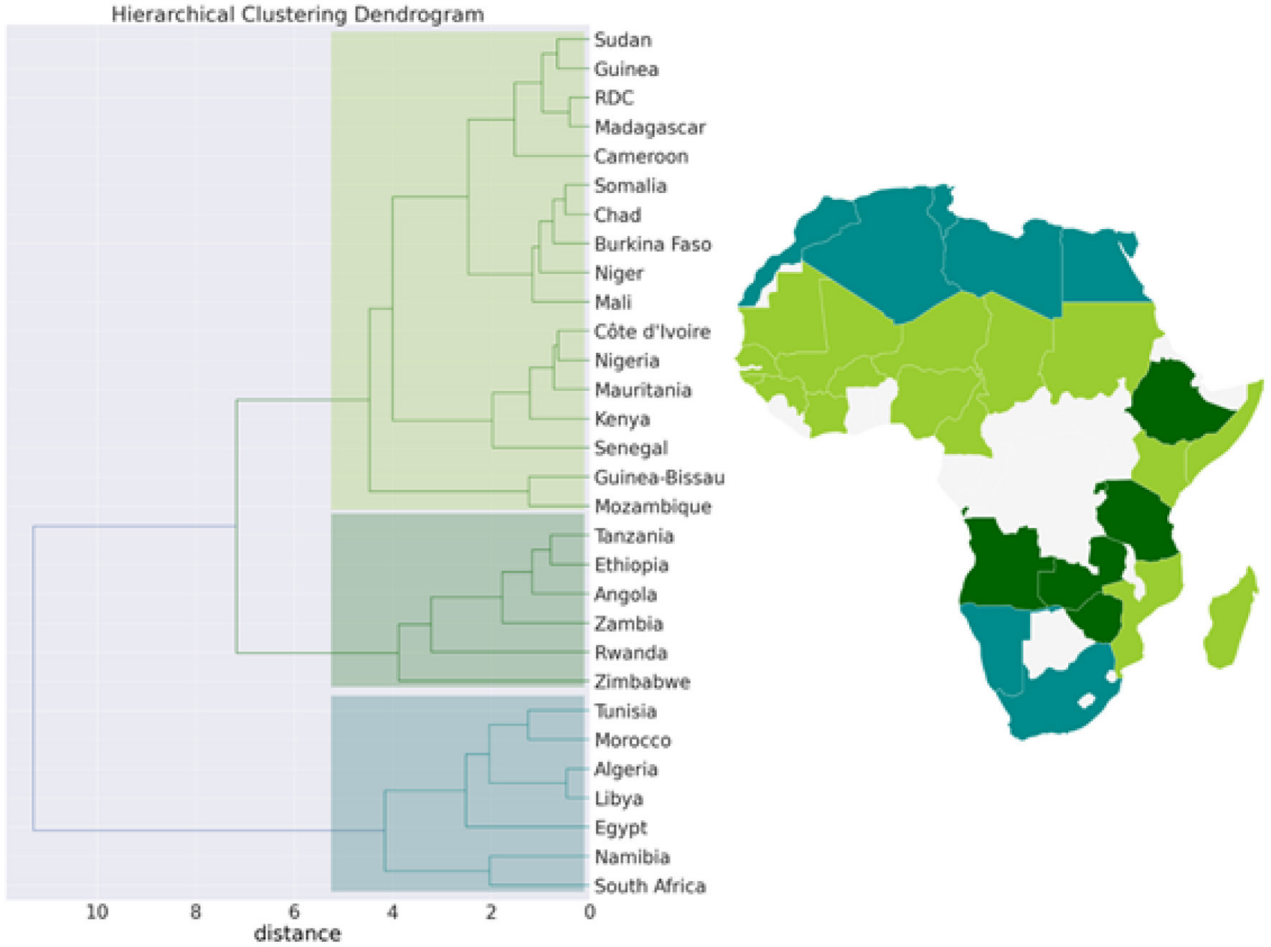
Hierarchical clustering (Dendrogram) for the pandemic, economic, demographic, and climatic variables allows countries to be grouped into 3 separate clusters.

**Figure 8:**
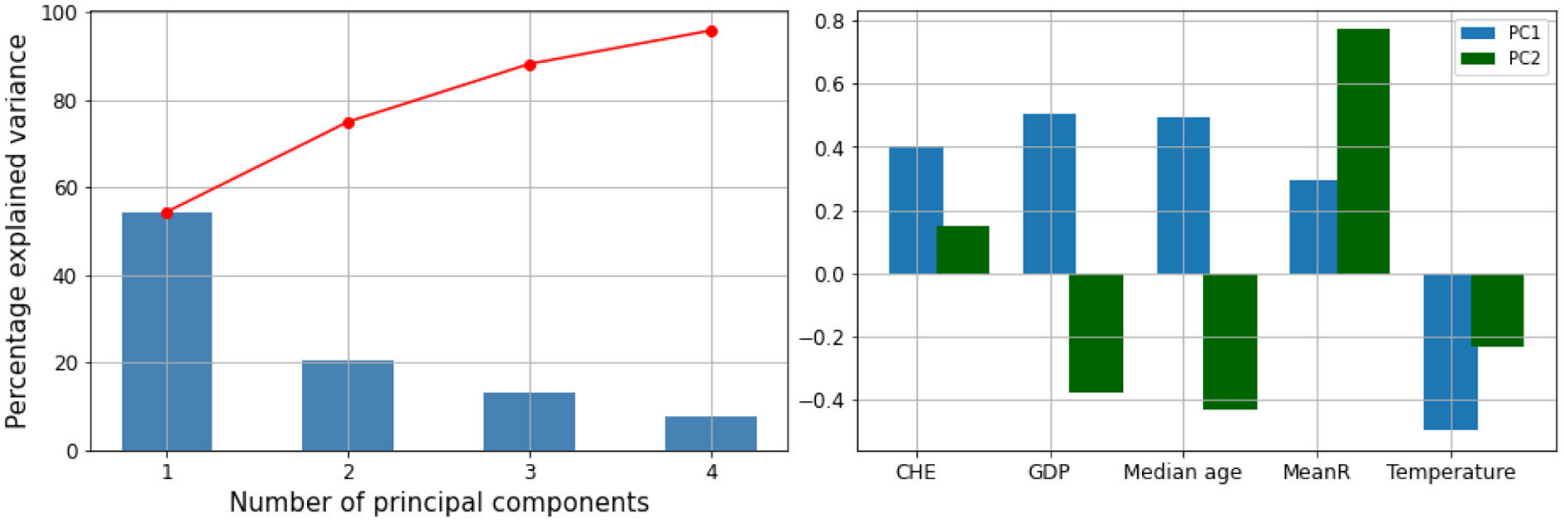
Principal components (PC) plot from the principal component analysis (PCA) on the pandemic, economic, demographic, and climatic variables.

**Figure 9:**
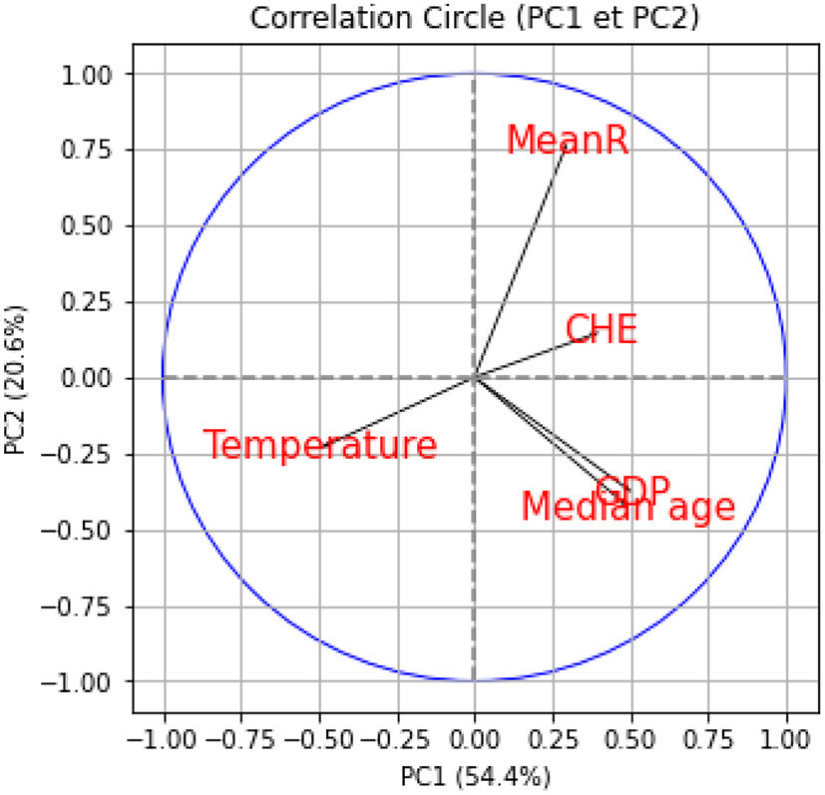
Correlation circles for the two first PC planes.

**Figure 10:**
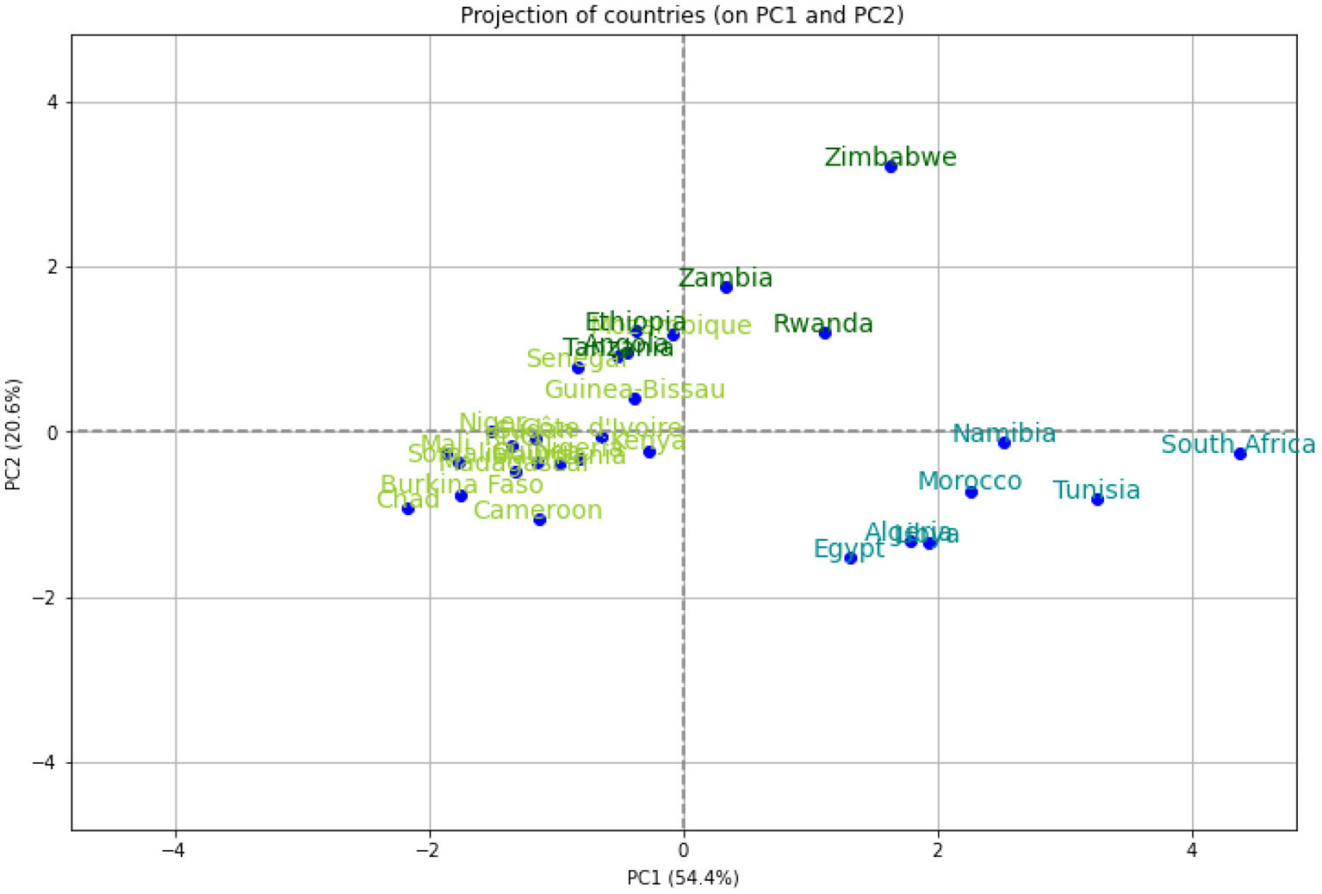
Projection of the 30 African countries of the PCA's plot on the first PC plane.

We performed a hierarchical clustering and a principal component analysis (PCA) (Figures 7–10). The PCA depicts two primary axes (PC1, PC2) that together account for 75% of country variation, 54.2% for the PC1 and 20.6% for the PC2. The primary parameters in PC2 are *MeanR*, median age, and *GDP*. For PC1, the primary parameter is the temperature.

We then performed a Hierarchical Clustering of the countries (see Figure 7). We were able to divide the countries into two distinct clusters. Morocco, Algeria, Tunisia, Libya, Egypt, Namibia, and South Africa make up the first cluster, which spans north and south Africa. This cluster is characterized by high median age and a high *GDP*>*US$*3000) (in [21.8, 32.7] years old). Except for Egypt (*MeanR* = 1.8), this cluster has witnessed an average of *MeanR*>2.2. We have identified five countries that had a significant first wave.

The second cluster is in turn divided into sub-clusters, the first sub-cluster includes Tanzania, Ethiopia, Angola, Zambia, Rwanda, and Zimbabwe. This cluster is characterized by high values of *MeanR*. *MeanR* in [2.4, 3.21] in Zimbabwe, a middle *GDP* in US$ [797, 1800], and a median age < 20 years.

The latest cluster includes the rest of the countries. This cluster is characterized by a high annual temperature of more than 25°C and a *GDP* of between US$2350 and US$438. These countries are distinguished by *MeanR* values in [1.69, 2.59]. Twelve of the 18 countries of the second Group are included in this cluster.

## 4. Conclusion

The objective of our paper was to document the Spatio-temporal variations in the baseline reproduction rate *R*_0_ and to understand the reasons for the different disparities between them. We highlight that more developed countries experienced a higher incidence in the first wave, which can be explained by their higher international exposure. We also show that the quality of health systems played a key role in limiting virus-related mortality. Consistent with the literature, we also show that countries with younger populations were less affected by the pandemic. Finally, we show that climate also plays a determining role in explaining the reproduction rate *R*_0_. At the end of the analysis of the determinants, we have made a clustering of the countries in order to identify which ones have been the most suffering during this pandemic or on the contrary which ones have been the most resistant.

Our results show that the geography of the pandemic in Africa largely overlaps with the geography of the wealth of the states. Consequently, the fight against poverty and the development of health infrastructures are sine-qua-non conditions for an effective fight against future epidemics or pandemic crises that could occur.

## Data availability statement

The original contributions presented in the study are included in the article/Supplementary material, further inquiries can be directed to the corresponding author/s.

## Author contributions

BN, AK, and SBe contributed to conception and design of the study. BN and WB organized the database. BN and SBe performed the statistical analysis. BN, SBo, and WB wrote the first draft of the manuscript. BN, SBo, AK, and SBe wrote the final draft of the manuscript. All authors contributed to manuscript revision, read, and approved the submitted version.

## Funding

This work was supported in part by the French Ministry for Europe and Foreign Affairs *via* the project REPAIR COVID-19-Africa coordinated by the Pasteur International Network association, by European Union's Horizon 2020 research and innovation program under grant agreement No. 883441 (STAMINA) and the Tunisian Ministry for High Education *via* PRF project PRFCOV19-D5P1 Evaluer, prédire, agir: stratégie fédérée pour la lutte contre la COVID-19.

## Conflict of interest

The authors declare that the research was conducted in the absence of any commercial or financial relationships that could be construed as a potential conflict of interest.

## Publisher's note

All claims expressed in this article are solely those of the authors and do not necessarily represent those of their affiliated organizations, or those of the publisher, the editors and the reviewers. Any product that may be evaluated in this article, or claim that may be made by its manufacturer, is not guaranteed or endorsed by the publisher.

## Footnotes

1 https://www.who.int/director-general/speeches/detail/who-director-general-s-statement-on-ihr-emergency-committee-on-novel-coronavirus-(2019-ncov)

2 https://www.afro.who.int/news/covid-19-cases-top-10-000-africa, https://covid19.ncdc.gov.ng/

3 https://www.afro.who.int/news/new-who-estimates-190-000-people-could-die-covid-19-africa-if-not-controlled

4 https://africacenter.org/spotlight/mapping-risk-factors-spread-covid-19-africa/

5 https://data.worldbank.org/indicator/SH.XPD.CHEX.GD.ZS

6 http://www.climatemps.com/

7 https://climateknowledgeportal.worldbank.org/country/central-african-republic/climate-data-historical

8 https://ourworldindata.org/grapher/daily-tests-per-thousand-people-smoothed-7-day

## Appendix: Model description

This model consists of the following system of ordinary differential equations:

(1)
$$\left\{ \begin{array}{l} S^{\prime }(t)=-\tau S(t)[I(t)+U(t)]\\ I^{\prime }(t)=\tau S(t)[I(t)+U(t)]-\nu I(t)\\ R^{\prime }(t)=\nu_{1}I(t)-\eta R(t)\\ U^{\prime }(t)=\nu_{2}I(t)-\eta U(t) \end{array}\right.$$

Where *t*≥*t*_0_ the time in days, *t*_0_ is the beginning date of each wave, *S*(*t*) is the number of individuals susceptible to infection at time *t*, *I*(*t*) is the number of infectious individuals at time *t*, *R*(*t*) is the number of reported infectious individuals at time *t* and *U*(*t*) is the number of unreported infectious individuals at time *t*. This system is supplemented by initial condition at time *t* = *t*_0_, (*S*_0_, *I*_0_, *R*_0_, *U*_0_).

We assume that the cumulative number of reported symptomatic cases at time *t* is proportional to the cumulative number of symptomatic cases for each time *t*. Let's denote the proportion coefficient by *f*. Therefore, the rate of asymptomatic infectious becoming reported symptomatic is ν_1_ = *fν* and the rate of asymptomatic infectious becoming unreported symptomatic is ν_2_ = (1−*f*)ν.

Table A1 represents the set of parameters that are fixed by the hypothesis and those evaluated by the country model.

**Table A1:** Parameters and initial conditions of the model 1.

**Symbol**	**Interpretation**	**Method**
*t*_0_	Time at which the epidemic started	Fitted
*S*_0_	Number of susceptible at time *t*_0_	Fixed
*I*_0_	Number of asymptomatic infectious at time *t*_0_	Fitted
*U*_0_	Number of unreported symptomatic infectious at time *t*_0_	Fitted
τ	Transmission rate	Fitted
$\frac{1}{\nu }$	Average time during which asymptomatic infectious are asymptomatic	Fitted
*f*	Fraction of asymptomatic infectious that become reported symptomatic infectious	Estimated
ν_1_ = *fν*	Rate at which asymptomatic infectious become reported symptomatic	Fitted
ν_2_ = (1−*f*)ν	Rate at which asymptomatic infectious become unreported symptomatic	Fitted
$\frac{1}{\eta }$	Average time symptomatic infectious have symptoms	Fixed

We assume that $\eta =\frac{1}{7}$ and $\nu =\frac{1}{7}$, are fixed for all African countries, which means that the average period of infectiousness of both unreported symptomatic infectious individuals and reported symptomatic infectious individuals and that the average period of infectiousness is 7 days. The fraction of total infectious cases that are reported *f* is unknown and varies from region to region.

The cumulative number of the reported symptomatic infectious cases at time *t* is obtained by using the following equation (21):

(2)
$$\begin{array}{l} CR\left(t\right)=\nu_{1}\int_{t_{0}}^{t}I\left(s\right)ds \end{array}$$

Since in the early stage of the epidemic, all the infected components of the system grow exponentially and the number of susceptible remains unchanged during a relatively short period of time *t*, we can fit an exponentially growing curve *CR*(*t*) to the cumulative reported cases data defined by the following special form :

(3)
$$\begin{array}{l} CR\left(t\right)=\chi_{1}\exp\left(\chi_{2}t\right)-\chi_{3} \end{array}$$

with χ_1_, χ_2_ and χ_3_ three positive numbers that we estimate using log-linear regression and the Genetic algorithm optimization method (35).

Following (21), we have:

(4)
$$\{\begin{array}{ccc} t_{0} & = & \frac{1}{\chi_{2}}(\ln(\chi_{3})-\ln(\chi_{1}))\\ I_{0} & = & \frac{\chi_{1}\chi_{2}\exp(\chi_{2}t_{0})}{f\nu }\\ U_{0} & = & \frac{\nu_{2}}{\eta +\chi_{2}}I_{0}=\frac{(1-f)\nu }{\eta +\chi_{2}}I_{0}\\ \tau & = & \frac{\chi_{2}+\nu }{S_{0}}\frac{\eta +\chi_{2}}{\nu_{2}+\eta +\chi_{2}}\\ R_{0} & = & \frac{\tau S_{0}}{\nu }(1+\frac{\nu_{2}}{\eta }) \end{array}$$
