# Supplementary material for: Spatio-temporal evolution of the COVID-19 across African countries
Source: Front Public Health. 2022 Nov 28;10:1039925. doi: 10.3389/fpubh.2022.1039925 (PMC9742427; doi:10.3389/fpubh.2022.1039925)
Supplement: Supplementary file 1 [file Data_Sheet_1.PDF]

Supplementary Material

1 DATA

Table S1. Economic, Demographic, Pandemics and Climatic Parameters

Country	CHE	GDP	Median age	Mean R	Mean T
Mozambique	7,834	448,544	17,6	2,33	23,95
Mauritania	3,301	1701,991	20,1	2,18	27,035
Senegal	4,132	1471,831	18,7	2,60	28,3525
Nigeria	3,027	2097,092	18,6	2,16	25,4375
Libya	6,05	3699,295	29,4	2,21	24,0975
Cameroon	3,595	1537,13	18,6	1,73	24,335
Egypt	4,74	3569,207	24,6	1,85	20,215
Rwanda	6,411	797,856	20	2,31	16,45
Madagascar	3,687	471,491	19,9	1,91	25,01333333
Kenya	4,592	1878,581	20,1	2,15	24,6
South Africa	9,109	5655,868	27,6	2,54	18,1875
Guinea	3,984	1194,038	19	2,04	26,3575
RDC	3,536	543,95	18,8	2,01	25,035
Ethiopia	3,238	936,34	19,5	2,63	23,3875
Chad	4,354	659,27	16,6	1,70	28,39
Côte d'Ivoire	3,299	2325,724	18,9	2,32	26,4275
Sudan	4,574	486,419	19,7	2,08	26,265
Mali	3,894	862,453	16,4	1,99	27,905
Niger	5,669	567,67	15,2	1,89	25,8875
Guinea-Bissau	8,35	727,52	18,8	2,18	27,315
Morocco	5,308	3058,692	29,5	2,29	19,7725
Algeria	6,243	3306,858	28,9	2,12	23,4725
Tunisia	6,958	3521,592	32,7	2,38	19,74
Angola	2,533	1776,167	16,7	2,49	22,06
Somalia		438,255	16,7	1,85	27,38
Tanzania	3,828	1076,47	18	2,43	23,21666667
Zambia	5,312	985,132	17,6	2,62	20,56
Namibia	8,497	4179,278	21,8	2,25	19,72
Zimbabwe	7,704	1214,51	18,7	3,22	20,6075
Burkina Faso	5,465	857,933	17,1	1,77	28,60666667

Table S2. Pandemics and Climatic Parameters

**Country**	R_1	T_1	$t0_1$	R_2	T_2	$t0_2$	R_3	T_3	$t0_3$
Mozambique	1.43	21.7	4/11/2020	2.42	26.5	12/28/2020	2.26	20.89	6/8/2021
Mauritania	1.4	33.4	5/21/2020	2.36	19.5	11/18/2020	1.52	33.78	6/22/2021
Senegal	2	33.3	3/3/2020	2.48	25.9	11/12/2020	2.55	29.06	6/15/2021
Nigeria	1.63	26.2	4/9/2020	2.29	23.4	11/28/2020	1.59	26.98	6/30/2021
Libya	1.36	22.7	3/24/2020	1.9	30.4	7/10/2020	2.57	30.39	6/23/2021
Cameroon	1.46	25.42	3/1/2020	2.02	24.15	11/24/2020	1.62	23.61	7/27/2021
Egypt	1.8	22.5	2/14/2020	2.48	14.4	12/11/2020	1.62	30.34	7/23/2021
Rwanda	1.54	16.9	5/31/2020	1.9	17	12/1/2020	2.54	12.8	5/31/2021
Madagascar	1.78	23.9	3/20/2020	2.19	26.5	2/26/2021	1.75	24.64	10/28/2021
Kenya	1.36	23.31	2/24/2020	1.66	25.37	10/3/2020	1.8	26.28	2/18/2021
South Africa	3.25	13.5	3/9/2020	2.06	23.1	11/27/2020	1.76	15.1	5/7/2021
Guinea	1.43	28.4	4/30/2020	1.7	27.4	2/6/2021	2.39	24.98	6/29/2021
RDC	1.45	25.7	3/18/2020	1.65	25.2	11/11/2020	2.03	24.9	5/14/2021
Ethiopia	1.76	21.4	5/2/2020	1.81	27.8	1/18/2021	2.13	23.05	7/19/2021
Chad	2.18	33.2	4/15/2020	1.77	30	7/3/2020	1.14	21.97	11/2/2021
Côte d'Ivoire	1.71	28.2	3/5/2020	1.56	26.1	5/24/2020	1.96	25.27	12/27/2020
Sudan	2.66	30.6	4/6/2020	2.38	25.8	10/20/2020	1.63	26.56	3/10/2021
Mali	2.11	33	3/17/2020	1.86	27.8	10/31/2020	2.1	28.34	2/17/2021
Niger	1.79	31.8	3/30/2020	1.51	20.5	2020-11-05	1.27	30.78	9/7/2021
Guinea-Bissau	1.95	30.3	4/19/2020	1.53	27.1	1/6/2021	1.75	26.85	7/4/2021
Morocco	2.56	13.96	2/15/2020	1.97	27.08	7/18/2020	1.86	27.1	6/29/2021
Algeria	2.47	10.8	3/4/2020	1.74	31.69	6/2/2020	1.69	18.06	10/6/2020
Tunisia	2.55	12.1	2/29/2020	1.65	24.3	6/3/2020	2.5	15.12	12/17/2020
Angola	1.34	18.81	5/11/2020	1.69	23	3/17/2021	1.53	23.47	9/7/2021
Somalia	1.68	28.52	4/10/2020	1.41	27.39	8/5/2020	2.06	26.24	2/9/2021
Tanzania	2.48	23.02	4/6/2020	2.81	22.94	9/8/2021	1.99	23.69	12/15/2021
Zambia	1.41	20.31	5/4/2020	2.65	17.72	6/28/2020	2.09	23.9	12/6/2020
Namibia	2.21	15.02	5/10/2020	1.92	24.42	11/30/2020	1.97	15.02	5/30/2021
Zimbabwe	1.8	16.3	7/5/2020	3.72	24.66	12/18/2020	2.53	16.81	5/31/2021
Burkina Faso	1.8	31.61	3/12/2020	2.21	25.99	11/20/2020	1.31	28.22	10/20/2021

Table S2. Pandemics and Climatic Parameters (cont.)

**Country**	R_4	T_4	$t0_4$	R_5	$t0_5$
Mozambique	3.2	26.71	12/4/2021		
Mauritania	3.42	21.46	12/24/2021		
Senegal	3.35	25.15	12/24/2021		
Nigeria	3.12	25.17	12/4/2021		
Libya	2.99	12.9	1/13/2022		
Cameroon	1.8	24.16	1/1/2022		
Egypt	1.51	13.62	1/13/2022		
Rwanda	3.24	19.1	12/10/2021		
Madagascar					
Kenya	2.2	23.44	7/15/2021	3.72	12/10/2021
South Africa	3.08	21.05	11/14/2021		
Guinea	2.64	24.65	12/23/2021		
RDC	2.9	24.34	12/1/2021		
Ethiopia	4.8	21.3	11/11/2021		
Chad					
Côte d'Ivoire	4.05	26.14	12/14/2021		
Sudan	1.63	22.1	11/1/2021		
Mali	1.89	22.48	12/18/2021		
Niger	2.99	20.47	12/21/2021		
Guinea-Bissau	3.47	25.01	12/25/2021		
Morocco	2.78	10.95	12/6/2021		
Algeria	1.86	33.34	6/28/2021	2.85	1/10/2022
Tunisia	1.84	27.44	3/13/2021	3.34	12/27/2021
Angola	5.4	22.96	12/9/2021		
Somalia	2.23	27.37	12/7/2021		
Tanzania					
Zambia	2.73	20.31	5/14/2021	4.23	11/29/2021
Namibia	2.89	24.42	12/6/2021		
Zimbabwe	4.82	24.66	11/22/2021		
Burkina Faso					
